# The do’s, don’ts and don’t knows of establishing a sustainable longitudinal integrated clerkship

**DOI:** 10.1007/s40037-019-00558-z

**Published:** 2020-01-17

**Authors:** Maggie Bartlett, Ian Couper, Ann Poncelet, Paul Worley

**Affiliations:** 1 Education in General Practice, Dundee University School of Medicine, Dundee, UK; 2 Faculty of Medicine and Health Sciences, Ukwanda Centre for Rural Health, Stellenbosch University, Stellenbosch, South Africa; 3 Department of Neurology, University of California, San Francisco, CA USA; 4 Department of Health, GPO Box 9848, 2601 Canberra, Australian Capital Territory Australia

**Keywords:** Longitudinal integrated clerkship, Medical education

## Abstract

**Introduction:**

The longitudinal integrated clerkship is a model of clinical medical education that is increasingly employed by medical schools around the world. These guidelines are a result of a narrative review of the literature which considered the question of how to maximize the sustainability of a new longitudinal integrated clerkship program.

**Method:**

All four authors have practical experience of establishing longitudinal integrated clerkship programs. Each author individually constructed their Do’s, Don’ts and Don’t Knows and the literature that underpinned them. The lists were compiled and revised in discussion and a final set of guidelines was agreed. A statement of the strength of the evidence is included for each guideline.

**Results:**

The final set of 18 Do’s, Don’ts and Don’t Knows is presented with an appraisal of the evidence for each one.

**Conclusion:**

Implementing a longitudinal integrated clerkship is a complex process requiring the involvement of a wide group of stakeholders in both hospitals and communities. The complexity of the change management processes requires careful and sustained attention, with a particular focus on the outcomes of the programs for students and the communities in which they learn. Effective and consistent leadership and adequate resourcing are important. There is a need to select teaching sites carefully, involve students and faculty in allocation of students to sites and support students and faculty though the implementation phase and beyond. Work is needed to address the Don’t Knows, in particular the question of how cost-effectiveness is best measured.

## Definitions of Do’s, Don’ts and Don’t knows

**Do’s**: Educational activity for which there is evidence of efficacy
**Don’ts**: Educational activity for which there is evidence of no efficacy or of harms (negative effects)
**Don’t knows**: Educational activity for which there is no evidence of efficacy

## Introduction

The longitudinal integrated clerkship (LIC) is a strategy for clinical medical education which is increasingly employed by medical schools across the world. In a LIC, students participate in the comprehensive care of patients over time and in educational relationships with those patients’ clinicians. They meet the majority of the core curricular competencies across multiple disciplines simultaneously [1, 2].

The underpinning principle of LICs is continuity [3]. In a LIC, students take an active role in the care of patients and have continuity of relationships with those patients’ clinicians over time [1, 3]. In practical terms, this continuity means students having the opportunity to engage with specific patients in a variety of healthcare encounters and interactions as they follow them though their healthcare journeys for the duration of the clerkship. Alongside this, continuity of educational supervision, patient care, peer groups and context leads to effective learning relationships [3–8]. For teaching clinicians, it means greater job satisfaction and fewer tensions between teaching and service delivery [9, 10] compared with short block placements. The principle of continuity of relationships is aligned with the concept of a ‘community of practice’, a social construction in which people share endeavours over time. These endeavours include learning and as a result of contributing to the care of patients alongside more experienced people in the community, students develop a sense of meaning and professional identity [11, 12].

LICs were first established in the USA in the mid-20th century as a way of enhancing recruitment to community-based roles in underserved, predominantly remote and rural areas [1, 2]. Because of perceived educational benefits, they have spread to a variety of different settings including some where a student’s clinical base is a tertiary teaching hospital in a large city [2, 13, 14]. Worley et al. [2] describe a large increase in the number and variety of LICs since the turn of the 21st century and a doubling in the numbers between 2009 and 2016 to 54 programs over four continents. The number of LICs has continued to increase [15, 16] and they remain part of a strategy to increase the sustainability of remote and rural healthcare in both developed and developing countries by providing context-specific, community-engaged education designed to help address work force shortages [17].

Worley et al. [2] describe a large variation in the populations in which LICs are based, with 20% of programs being in settlements of 25,000 people or fewer and 24% in settlements of over 100,000. LICs fall into two broad models: a predominantly community-based model in which students are based in family medicine settings, often in rural or remote locations (the ‘dispersed immersed’ model [18]), and one in which students learn in secondary or tertiary care centres and are supervised by clinicians from multiple disciplines concurrently (the ‘parallel streaming’ model’ [18]).

Many LICs started as pilots with small numbers of students before scaling up to larger groups, but many have stayed small and exist as a voluntary alternative to a more traditional block rotation-based curriculum. A small number of institutions have a compulsory LIC for the whole cohort [19, 20].

Medical educators developing and running LICs often face multiple challenges; we are aware of at least two LICs that have not continued. While there is some published literature describing how institutions went about setting up their LICs, and by implication, some consensus about what is likely to give them the best chance of success, there is very little that specifically considers what is needed for a LIC to be sustainable in the sense that it can be maintained over time. This sustainability may comprise such programs’ acceptability to students, their learning experiences and performance, their achievement of curricular goals and the adequacy of their supervision. In broader terms, sustainability may also include impacts on the health needs of the community the LIC serves, and economic considerations such as cost-effectiveness [2].

Snadden et al. [21] consider the challenges of expanding teaching capacity by using new, often regional, locations. This paper is not focused on LICs but much of what it describes is relevant; they identify that building and maintaining relationships, supporting students, and developing teaching faculty all contribute to the sustainability of these new sites.

Experimental work on the question of sustainability is scarce and because the majority of existing LICs are relatively recently established, many do not have long-term outcomes data. Worley et al. [2] identified ‘the cost-effectiveness and sustainability’ of the LIC approach as relevant questions.

This paper reports on a narrative review of the published literature by a group of authors with 56 years of collective experience of establishing and maintaining LICs in four continents, giving guidance in the form of the Do’s, Don’ts and Don’t Knows of setting up a LIC with a view to its sustainability over time.

## Method

This is a narrative review of the literature which incorporates both published evidence and our own experience. We have emphasized evidence from the literature that has informed decisions in our own educational practice and have attempted to appraise each paper critically to assess the strength of the evidence underpinning each of our guidelines [22]. When making judgments about the strength of the contributing evidence, more weight was given to papers arising from the longest established LICs, as these have demonstrated sustainability over time.

Each author independently provided a list of the Do’s, Don’ts and Don’t Knows of establishing a sustainable LIC based on their experience and knowledge of the published literature. Each provided a list of the underpinning literature that they considered to be strongly influential on their own educational practice in the field of LICs. These were collated and compared by MB and the combined list was then shared with the rest of the author group for comment and discussion. Ancestry searching led to more papers, which were assessed for relevance and strength and included where they contributed to the evidence for the guidelines. Finally, a search for new items published in the 9 months between starting this review and submission of this paper was undertaken via EBSCO Medline using the search term ‘longitudinal integrated clerkship*’. We finalized the guidelines in electronic discussion after the synthesis of the papers selected for inclusion.

We provide a summary of the key evidence to support our recommendations, indicating the strength of evidence. Tab. 1 shows the criteria for strength of evidence [23] and Tab. 2 a list of the Do’s, Don’ts, and Don’t Knows.

**Table 1:** Criteria for strength of recommendation

Strong	A large and consistent body of evidence
Moderate	Solid empirical evidence from one or more papers plus consensus of the authors
Tentative	Limited empirical evidence plus the consensus of the authors

**Table 2:** The guidelines

		Strength of evidence
*Do’s*		
Guideline 1	Manage change proactively with a focus on building relationships	Moderate
Guideline 2	Clearly articulate and manage learning outcomes and expectations	Moderate
Guideline 3	Ensure enduring effective and responsive central and local leadership	Moderate
Guideline 4	Promote Communities of Practice [11, 12] in which students are co-providers of healthcare	Strong
Guideline 5	Select sites which are able to deliver the curricular outcomes and support students’ learning	Strong
Guideline 6	Develop strategies to recruit and retain skilled faculty	Strong
Guideline 7	Ensure effective and responsive support for students	Strong
Guideline 8	Ensure good initial and ongoing faculty development	Strong
Guideline 9	Ensure adequate resources	Moderate
Guideline 10	Evaluate systematically to identify problems and opportunities for development	Moderate
Guideline 11	Explicitly include the desired workforce outcomes in the design	Strong
*Don’ts*		
Guideline 12	Don’t restrict LICs to rural, regional, general practice or community-based settings	Strong
Guideline 13	Don’t underestimate the importance of alignment with assessment	Moderate
*Don’t knows*		
Guideline 14	Which students will do best on a LIC?	
Guideline 15	How is cost-effectiveness best measured?	
Guideline 16	What is the optimal structure in a LIC timetable?	
Guideline 17	How long should a LIC be?	
Guideline 18	What are the effects on patient care?	

## Results

### Do’s

#### Guideline 1: *Manage change proactively with a focus on building relationships* (Moderate)

The majority of clinicians currently involved in teaching medical students have learnt in a block rotation structure in which students move through a series of short discipline-based clinical blocks and, in the light of their own personal success, and perhaps wearing rose-coloured spectacles, may view it as the best way to provide clinical training.

LICs are very different from a block rotation-based clinical curriculum and implementing change even for a small cohort LIC that runs alongside an existing curricular structure is challenging. As with any proposed change, attention needs to be given to the process of change which starts with defining and then sharing a vision, followed by broad and enduring consultation with relevant groups, making decisions about the practicalities and then implementing, maintaining, evaluating and developing the changes [24, 25].

Resistance to change in a medical program can come from faculty, health services and regulators, and potentially from patients, students and their parents [26] even when a proposed change has strong voices promoting it and sound evidence behind it. As well as change itself being complex, a LIC is an innovative and complex system with many stakeholders, relationships and challenges. Once a change is made and a new program launched, it is not guaranteed that resistance will cease or that the change will be sustainable. Factors that can lead to the non-sustainability of a program include changes of leadership, funding, politics, regulation and curricula.

A number of papers describe how institutions went about managing a curricular change from a block rotation to a LIC [13, 14, 24, 27–31]. All included the need to overcome inertia and achieve buy-in from a wide variety of stakeholders including clinicians, academics, students and health communities. A significant aspect of this is the demonstration of benefits for all from having better trained doctors, more engaged students, involvement of new sites and supervisors reduced capacity pressures overall, increased rural and generalist recruitment, and fewer tensions between service delivery and teaching [14, 27, 32, 33].

Hudson et al. [34] describe the use of an existing framework for change management to support the implementation of a large scale, whole class LIC at the University of Wollongong in Australia. They argue that deriving a strategy from such a framework will help to achieve sustainability. An emphasis of the framework they used (Roberto and Levesque’s Change Management Framework [35]) is on relationship building, which is appropriate in the context given that the LIC model has relationships at its core [1, 4, 5, 36–44]. Hudson et al. [34] emphasized the importance of maintaining communication with all partners and stakeholders (supervisors, students, patients and healthcare providers) throughout and beyond the implementation and evaluation phases. Students, in particular, can be powerful advocates of curriculum change and should be included in the design process.

Though there is no one identifiable best approach, it is clear that establishing a LIC is a complex and disruptive process on both practical and emotional levels [1, 26], and careful ground work is needed before embarking on it. We conclude that a planned approach focused on building relationships is likely to give the best chance of sustainability.

#### Guideline 2: *Clearly articulate and manage learning outcomes and expectations *(Moderate)

Collaborative approaches to ongoing conversations between medical schools, health services and students are needed in order to reach a shared understanding about the expected outcomes of LIC programs for both students and the communities in which they learn, so that education and training can be aligned with these outcomes [45, 46]. For new programs that involve only a proportion of the whole class, learning outcomes may need first to map against existing curriculum outcomes. There will usually be additional outcomes specific to the LIC that need to be taken into consideration; these are often outcomes that make LICs attractive to students and communities and reflect reasons why the LIC was created in the first place. They may include addressing workforce needs, community engagement, professional identity formation, patient centredness, and being able to practice independently. Such additional outcomes should be formally evaluated if possible, but some are less easily measurable than others.

The unique features of LICs mean that some outcomes cannot be compared between the two. For example, where a dispersed, immersed LIC is located in a remote location, students’ experiences and learning will be very different from that of their peers in a parallel streaming LIC or a block rotation in an academic teaching centre, but no less valuable. The expectations of the outcomes of the programs should reflect and celebrate such contextual differences [47] rather than aim for standardization [48]. In our experience, attempts to replicate all outcomes across co-existing programs in one institution, or between institutions, threaten the sustainability of a LIC.

#### Guideline 3: *Ensure enduring, effective and responsive central and local leadership *(Moderate)

Sustainability depends on continuing leadership once an initial change has been implemented. At a local level, leadership must be visible, accessible, responsive and adaptable to changing local contexts [15]; effective leadership meets the needs of all those involved and mitigates instability that could threaten the LIC. This includes leading the community engagement that is critical to the LIC’s quality and long-term sustainability [46]. While in the early days all stakeholders need time to settle into a new curriculum, there needs to be ongoing program development that is partly reactive to issues identified through rigorous local evaluation and partly receptive to an evolving evidence base in the field.

The need for local champions has been identified [49–51], particularly to work with colleagues in the setting which is not a student’s main clinical base (secondary care for those who are based in primary care, and vice versa). This role promotes the LIC and disseminates information amongst clinicians who do not have a formal educational role but who will encounter students as they co-provide care for patients. In parallel streaming models [2, 18] a champion within each discipline is critical.

New leaders must be identified, developed and nurtured by active engagement with those involved in delivering LICs. Succession planning is important for the sustainability of a program. We are aware of two programs in which the loss of an established leader threatened the programs, one of which ceased to exist ([26], Jones S—personal communication 2019). It is not only losses of the LIC leaders themselves that may threaten sustainability; changes in the levels of leadership above may also have an impact, for example where higher levels of leadership change from being supportive of a LIC to being unsupportive. For a LIC to be sustainable, therefore, there needs to be an awareness of the risks and continuous and intentional management of them at all levels of leadership. LIC leaders need to work with successive institutional and health service leaderships to meet the overall goals of curricula, service delivery and workforce planning while maintaining the ethos and principles of the LIC.

Part of the role of the local leadership is to support the development of a ‘constellation of communities of practice [11]’ in which the LIC can function. This includes postgraduate programs, and it is helpful if there is parallel and synergistic development of both so that there is alignment of values and principles in leadership and faculty development [49]. A similar alignment is needed with local health service leaders.

Leadership at a global level can support LICs by driving creativity, developing and disseminating resources and scholarship and sharing good practice. The international Consortium of Longitudinal Integrated Clerkships fulfils the function of a global community of practice for LIC leaders [15].

#### Guideline 4: *Promote communities of practice in which students are co-providers of healthcare *(Strong)

The continuity of a LIC enables the development of relationships within communities of practice, giving students opportunities to see, and engage with, the continuum of illness and healthcare and the outcomes of clinical decision making [3]. Having an active role in the provision of healthcare for patients with whom they have developed a relationship over time leads to ‘an ethic of caring’ [52]. This helps with professional identity development [11] and promotes learning as patients and communities start to matter to students [53, 54].

A number of papers describe the enhancement of students’ learning by being actively involved in the care of patients [38, 41, 52, 54–59] and part of this is an effect of the responsibility students must take on in LIC programs [52]. Prideaux et al. [38] use the word ‘symbiosis’ to describe mutually beneficial relationships between medical schools and health services, and also between students, clinical educators and patients. Halaas et al. [54], commenting on the success of Minnesota’s long running LIC, describe students practising alongside their preceptors, many of whom are graduates of the same program which must lead to a powerful sense of continuity and belonging.

Hauer et al. [41] compared the perceptions of students on block rotations and LICs in three USA medical schools. They concluded that while both groups perceived a role for themselves in patient care, the LIC enabled students to become highly integrated into healthcare teams and to experience progressive independence based on the continuity of relationships within those teams. This resulted in them reaching higher levels of performance and satisfaction with their contributions than those on block rotations; the LIC students described a ‘doctor role’ for themselves. Steven et al. [57] analyzed audio diary entries of students with the aim of exploring how students learnt from real patients during clinical clerkships (though not specifically LICs). The authors concluded that students’ learning in the clinical setting is enhanced when clinicians actively share their expertise in dialogue and engage students in co-participation in patient care in communities of practice. Van Schalkwyk et al. [58] describe similar findings in a South African LIC.

As well as the benefits to students that are associated with learning in a community of practice, there are benefits to the communities of practice. Hudson et al. [20] describe the ‘transformation’ of a rural community of practice in Australia, with an increase in morale and enthusiasm amongst healthcare providers more widely than only amongst those actually teaching. They ascribe this partly to the students’ contribution to the work of the teams, but also to the empowerment arising from the relationship with the university, the affirmation of realizing that they could offer high quality teaching and the recognition that it could improve recruitment of doctors to the area. A student on this program identified the students’ role in achieving its sustainability in that they needed to engage with preceptors by discussing their own educational needs, actively contributing to clinical work and giving the preceptors feedback. This reinforced preceptors’ engagement and satisfaction. Students’ induction to the programs should encourage them to engage with their preceptors in this way.

An evaluation of two rural LIC programs (one in Australia and one in Canada) identified that local community leaders, local government officials and health service managers saw a broader value than health-related outcomes for their communities, citing, for example, the importance of the programs for future workforce recruitment, for retention of existing healthcare staff, for local economic development and for offering hope to rural youth [49]. For a small English town (population around 10,000) there was a strong sense of civic pride in having links with a medical school via its rural campus [30], which supported ten students learning in an amalgamative clerkship (one in which an extended placement forms part of an academic year’s curriculum but does not fully meet the criteria of a LIC according to Worley et al. [2]). The Northern Ontario School of Medicine is proactive in maintaining their distributed teaching sites’ involvement in medical education by engaging with civic leaders and communities to introduce new LIC students at the start of each academic year (R Strasser—personal communication, 2019).

Patients are part of a LIC and the involvement of students in their care over time must be perceived by them as acceptable and even beneficial. There is evidence that this is the case [55, 60, 61]. For a LIC to be successful and as a result to be sustainable, the making of connections between all relevant groups, including patients, should be promoted and supported to create functional and enduring social learning systems, or communities of practice [11, 62, 63].

#### Guideline 5: *Select sites which are able to deliver the curricular outcomes and support students’ learning *(Strong)

Norris et al. [1] specifically note the need for ‘careful site selection’ and Couper et al. [49] describe it as a ‘critical issue’ when introducing this type of program. It involves a consideration of the ability of a site to deliver opportunities for students to meet all of the year’s curricular outcomes (capacity, local epidemiology and resulting caseloads, range of services and facilities) and to meet their social and practical needs (accommodation, travel, leisure activities, peer groups), as well as provide effective support for teaching in terms of faculty (size, skills and commitment), and administrative and information technology infrastructure.

The majority of the early LICs arose out of the need to improve recruitment to regional, rural and remote locations [1, 2]. This naturally led to them being located in less populated, less well-resourced areas, as opposed to the large tertiary care or academic teaching centres in which medical education has historically taken place. More recently, the LIC model has been imported into tertiary and academic centres in which the principles of continuity and symbiosis [4, 38] are being successfully applied to these environments [2, 13, 14, 64].

In some LIC programs, all of the students are hosted in one location such as in the Harvard program [64]. In others, such as the Northern Ontario School of Medicine in Canada [19] and Flinders in Australia [65], students are placed in one of several widely distributed sites with varying characteristics. Couper et al. [49] comment that this variation between sites is appropriate, that different sites are suited to different students and that matching students to sites is important, although there is no guidance about how this might be done. To achieve sustainability, there needs to be flexibility within a LIC program so that it can be adapted to work in distributed sites with different characteristics [49, 51] and to make best use of the site’s strengths [28].

There are challenges for students linked to the distance of distributed sites from the main campus. These are focused on social isolation, the lack of familiarity with the location, driving safety and technological links [30, 49]. For the majority of LICs, remoteness and rurality are a fundamental feature and therefore it is managing these challenges and the expectations of students, and where possible giving students informed choices about where they are placed, which will lead to sustainability rather than the location of the site itself.

#### Guideline 6: *Develop strategies to recruit and retain skilled faculty *(Strong)

It is necessary to recruit excellent and committed clinical teachers to any clinical educational program and then retain them. However, there is a specific challenge in doing so in the settings in which LICs often exist, where workforce shortages have been a driver for their establishment and where resourcing teaching in terms of extra clinical staff is difficult [51, 66–68].

Clinical teachers are likely to have experienced tensions between service delivery and teaching, which is often a barrier to teaching. There is convincing evidence that these tensions may be reduced in LICs as students contribute to patient care and the longitudinal relationships between teachers and students are satisfying to both [1, 33, 51, 66, 69]. This information can be used in recruitment activities.

Another challenge for clinical teachers in a LIC program is the burden of responsibility arising from their perceived sole responsibility for students’ learning in a particular discipline [66], in general [70], or in a primary care base [51]. This can be reduced by ongoing communication between central faculty and clinical teachers and an emphasis on the role of the communities of practice in which the students are learning.

Part of retaining clinical teachers is having an awareness of the challenges they face, responding promptly when there are problems and maintaining flexibility of expectation.

Christner et al. [67] recommend that ‘medical schools develop ways to meaningfully reward community-based faculty’ which could include formal and public recognition, financial incentives and novel opportunities for continuing medical education. There should be a focus on making the contributions of clinical teachers ‘visible and rewarded’ [71]. Some medical schools have developed higher degrees for those clinical teachers who wish to be more deeply involved in and engaged with medical education, leadership and research, such as the Masters in Clinical Education program at Flinders University (Australia) [72]. The University of California San Francisco involves community-based LIC directors in its Academy of Medical Educators which is a formal structure which makes then eligible to participate in faculty development programs [73]. For many clinical teachers, being explicitly part of a medical school’s community of practice [11] is important and by supporting and encouraging this, we can strengthen local expertise, confidence and leadership.

#### Guideline 7: *Ensure effective and responsive support for students *(Strong)

Students need support in navigating transitions, clarifying roles and tasks, managing interpersonal challenges and social and educational isolation [8]. Where students learn in distributed settings, such isolation occurs as a result of them moving away from pre-existing social networks and peer groups. Sustainability of a LIC may be threatened if this isolation is not considered and addressed. Selection of students in the first place may be part of this, but it is important that central and distributed faculty are aware of potential isolation and that supportive measures are in place. Couper et al. [49] recommend that students should be prepared to feel isolated at times, and that faculty actively manage their expectations around this, which includes normalizing it.

Daly et al. [62] describe students’ geographical separation from their networks as a ‘double-edged sword’; the students may be socially isolated but this helps them to develop a sense of belonging to their LIC communities. In PW’s experience at Flinders, the students who were placed close to their university tended to travel back much more frequently, or to commute to their placements. This became a burden for some of them and their learning was compromised by not being as immersed in their communities.

Alongside experiences of isolation, students experience uncertainty about the progress of their learning, and this often happens early in the clerkship when they have moved from a more structured teaching environment and are getting used to a more self-directed approach. Clinical teachers’ awareness of this transition and the challenges it causes for students will enable them to be proactive in orienting and supporting students in the new way of learning [19, 45, 68, 73].

Despite faculty awareness and attempts to forewarn students and manage expectations, the challenges of social isolation and the different approach to learning that students experience when they move into a LIC can be very difficult, especially as students have a tendency, in our experience, to over-estimate their resilience. Such challenges, if successfully overcome, are examples of the ‘desirable difficulties’ [75] that lead to learning and can increase students’ self-efficacy for future practice [76] and of the disorienting dilemmas that lead to transformative learning [77, 78].

While it is recognized that LICs provide the opportunity for students to be individually mentored, guided and coached by clinicians in a way that is often lacking in large academic centres [79], they may face the same life challenges as their colleagues (for example relationship difficulties, personal or family illness, bereavement) without their usual level of family or community support. Programs need to ensure systems are in place to provide support and focused interventions for such students; often systems are in place centrally but their application on a distributed platform is not considered.

Work by Cuncic et al. [39] suggests that preceptors should be encouraged to develop trusting relationships with students, including friendship, advocacy and concern for their emotional well-being. Social interactions outside of the clinical environment help with this, particularly where preceptors are able to view the students as junior or future colleagues. By forming individually focused relationships, preceptors’ approaches to teaching and the provision of personal support, beyond that required for the meeting of educational outcomes, can lead to students’ personal growth.

The beneficial relationships and social interactions between preceptors and students are not confined to LICs in small communities; there is evidence [64, 69] that demonstrates their importance in LICs which are based in urban centres. Two author groups report expressions of concern about professional boundaries and the risks of them being crossed [39, 51]. However, both comment that this is not usually a problem in practice; Cuncic et al. [39] talk about the familiarity rural doctors have with navigating such boundaries in small communities, and in Dundee’s LIC, it was a concern for tutors in advance of the LIC starting, but was not borne out in practice [51]. The students on two programs in the UK valued and enjoyed the social connection they made [42, 51].

Effective communities of practice (see Guideline 4) inherently provide support for all members and so their development needs to be promoted and supported by central faculty and healthcare providers. It is likely that LIC students are involved with multiple communities of practice, some with tensions between them [43, 62]. Daly et al. [62] suggest that students should be supported to recognize communities of practice and to navigate the boundaries between them.

In terms of the support needed for students to meet their educational outcomes, skilled mentorship is needed from preceptors and tutors who are knowledgeable about the learning objectives of the curriculum and are able to promote students’ development by supporting them in progressively more complex responsibilities over time and giving them useful, tailored feedback [3, 10, 28, 69]. Students need more intensive support in the early weeks of a LIC but that need reduces over time [19, 51].

Oswald et al. [26] recommend that small stable groups of students work together as they learn from and support each other and von Pressentin [45] recommends that ‘action learning sets’ (which include students, educators and LIC leads) at all LIC sites in a program are set up in which all learning outcomes can be developed and navigated. This means that all participants share understanding of the processes and outcomes of the LIC. Continuity peer groups in the clerkship year, including in LICs, provide anticipatory guidance about clinical expectations, best practices in interacting with patients and supervisors, and information about implicit rules of clerkships [8].

#### Guideline 8: *Ensure good initial and ongoing faculty development* (Strong)

To be sustainable, any educational program must have enough committed and enthusiastic teachers with appropriate skills to support learners. The key skills of clinical teachers in a LIC are supporting students to identify their own learning needs in the context of taking responsibility for patient care and giving useful, incrementally progressive feedback. In clinical educational settings, role modelling is an important facet of teaching [80, 81].

A new initiative will inevitably involve the need for extensive and vigorous faculty development tailored to the needs of the program, and as a LIC represents a significant move away from a very established and widely embedded model of clinical education, this need is all the greater. As well as developing teachers’ skills, there is a need to encourage the letting go of old ways of doing things [13, 20, 27, 68].

Blitz et al. [82] describe the importance of educators at distributed sites feeling that they are part of the medical school with roles to play in curriculum development. They wanted feedback to help them develop as educators and saw trusted colleagues as resources for their own learning [82]. Zelek and Goertzen [83] suggest that clarification of the intrinsic and extrinsic motivators of distributed clinical teachers can help central faculty to engage them in teaching over time and thus sustain such distributed programs.

A key concept to be addressed in the faculty development associated with LICs is that of ‘coveritis’—the tendency of clinical teachers to want to hand all their knowledge on to learners by covering everything often in a didactic manner [13, 84]. This concept is completely counter to the LIC principles of students identifying their own learning needs and seeking out their own opportunities to learn arising from individual encounters with patients.

With regard to the specific question of how faculty are trained to undertake workplace-based assessments in longitudinal settings, Dory et al. [85] note that assessors are ‘notoriously inconsistent’ and while this is often mitigated by using multiple assessors, the continuity of supervision inherent in a LIC may reduce this effect. They found that assessors’ behaviour was influenced by beliefs about generic competencies; whether they are fixed attributes or ones that can be influenced by teaching. Dory et al. [85] conclude that assessor values and resulting behaviours must be understood in order to influence them.

In the context of communities of practice, faculty development should transcend the boundaries of specialty and role, and be a collegiate activity in which the development of relationships throughout the community is emphasized [39, 49, 80]. It is important too that faculty development is not simply focused on training clinical trainers to perform their teaching tasks but also very much on supporting them in their dual roles as clinicians and teachers, or, particularly in rural areas, in the multiple roles they play [44, 69]. It is vital that faculty development occurs within the context of building mutually beneficial relationships.

#### Guideline 9: *Ensure adequate resources *(Moderate)

LICs are known to be logistically complex and organizationally demanding [1, 26]. This complexity arises partly from the need to ensure continuity of patient care, supervision, peer groups and curricula and partly as a result of the underpinning principle that students’ learning is independent and linked to the patients they encounter. Inevitably, there are resource implications in terms of leadership, administrative staffing, capacity in both clinical services and physical space for teaching, faculty development and remuneration, and information technology infrastructure (in both teaching spaces and students’ living accommodation) [14, 20, 26–28, 33, 49, 51, 69, 86].

Given the distributed nature of many LICs, information technology infrastructure must be reliable and fit for purpose, particularly in that different systems must interact with each other across education and health service boundaries [49, 51]. Electronic methods of communication are very useful for keeping in touch with students and faculty, reducing isolation and providing educational sessions and resources [87, 88].

In terms of the resource implications for an individual practice, Walters et al. [89] explored the cost of a LIC in terms of consultation length, and found that it did not increase for preceptors when supervising students in a parallel consulting model (students have their own consulting room and see patients alone at first, then the preceptor joins them to conclude the consultation). They concluded that this model of precepting contributed to the program’s sustainability. It could therefore be recommended in LIC programs, though with the proviso that teaching sites must have adequate rooms available.

#### Guideline 10: *Evaluate systematically to identify problems and opportunities for development* (Moderate)

As with any curriculum, either recently introduced or long established, a systematic program of relevant and comprehensive evaluation is needed to consolidate and develop it [24], and to build a body of evidence that it is functioning well to deliver its intended outcomes. Such evaluation should address all of the key performance indicators. Inviting experts to undertake these evaluations on a regular and frequent basis not only gives the benefit of their wisdom, but it can help to demonstrate the value and significance of the program to university leaders and external funders.

In a field that is relatively new and rapidly developing, program leaders must keep abreast of developments so that their LIC can be refreshed and updated with the benefit of the experience of others, the sharing of best practice and the evidence they provide for change. Developing and leveraging a culture of innovation can help support ongoing iterative improvement and change for the LIC. However, the possibility of ‘change fatigue’ needs to be borne in mind.

As well as ongoing evaluation, ideally a LIC should have a formal research program to develop legitimacy within the institution and to apply academic rigour to the LIC concept. However, this would require resources in terms of time and training. The Consortium of Longitudinal Integrated Clerkships, as an international research collaborative, seeks to develop a cross-institutional evidence base which may help to develop such legitimacy and rigour [2].

#### Guideline 11: *Explicitly include the desired workforce outcomes in the design *(Strong)

For many LICs, a key goal is workforce transformation. This may be redistribution to rural areas [90], promotion of ambulatory care [14], or improvement in patient centredness and idealism [91]. For some LICs, such goals are explicit in funding to support the innovation [46]. Whilst there is strong evidence supporting the potential of LICs to achieve these outcomes, these aspirations are not intrinsic to every LIC, and explicit consideration is required to maximize the desired impact of a particular LIC on subsequent student careers.

For example, in the case of promoting rural practice, there is strong evidence to support including in the LIC design preferential selection of rural origin students [92], basing the LIC in rural practice for at least 12 months [93], and linking the LIC with rural postgraduate training opportunities [94, 95]. Similarly, in relation to patient centredness and idealism, the selection of supervisors who will support and model these attributes is important, as is discretionary time in the curriculum to support the student pursuing continuity in clinical relationships with patients [13].

### Don’ts

#### Guideline 12: *Don’t restrict LICs to rural, regional, general practice or community-based settings *(Strong)

Though most of the early LICs were based in rural regions with workforce challenges [1, 2], the literature reports on successful LICs based in larger urban centres and tertiary teaching hospitals [13, 28, 64]. The number of non-rural medical schools which have, or are planning, a LIC within their programs continues to increase [15, 16]. For many of these, the reasons for introducing a LIC are increasing continuity for students both in terms of relationships with patients and educational supervision and teaching [15] with the implication that it is believed to enhance learning rather than being a means for recruiting students to a specific career path.

#### Guideline 13: *Don’t underestimate the importance of alignment with assessment *(Moderate)

Medical schools rightly expect that all graduates meet the graduate attributes and required knowledge skills and attitudes, irrespective of whether they learn these in a LIC or a rotation-based system [96]. However, how and when students are assessed can either support or undermine a LIC. Assessment drives learning [97], thus congruence between the assessment and curricular programs is just as important for LICs as for older approaches.

For example, a medical school may have chosen to assess each clinical discipline separately and sequentially at the end of each 6‑week rotation throughout the year, so that there is an examination in surgery at the end of the surgical block, one in paediatrics at the end of the paediatrics block and so on. Following this pattern for the LIC students will result in the LIC students being disadvantaged because the LIC is based on students acquiring their learning in each discipline in parallel over the entire LIC period, not just the first 6 weeks, and the students will understandably focus almost entirely on their surgical texts for the first 6 weeks, the paediatrics for the next 6 weeks etcetera and not take the opportunities for integrated learning that are the hallmark of LICs.

Creating a bespoke assessment process for the LIC may appear initially to be another alternative, but this opens up the LIC, its students and teachers, to criticism of unequal, and potentially lower, outcomes and performance. LIC programs have demonstrated that students can be assessed fairly using the same assessment items used by rotation-based curricula, but the timing of these assessments should be at the end of the LIC [65]. More preferable is using the LIC to develop integrated assessments across disciplines for all students and including the LIC faculty in the creation of these whole of school assessments [98]. The focus across the program should be on equivalence rather than replication of assessment, so that LICs are able to maximize assessment for learning in context. This is based on the underlying principle of many roads leading to one destination [6] that is the assessment of common outcomes and not on the routes to achieving these.

### Don’t knows

#### Guideline 14: *Which students will do best on a LIC?*

In order to be sustainable, programs need to attract students and meet their social and learning needs. Many LICs are voluntary within an undergraduate medical program and most have formal application and selection procedures. These should be designed to ensure a good fit between students and their learning environments [1, 54, 99]. Where LICs are voluntary, studying the characteristics of those who choose a LIC may help to define those who will do best, but with the risk of producing unjustified rules that exclude others who, for a variety of practical reasons, have not chosen a LIC but who might have done very well if they had. There is debate about whether academically weaker or stronger students would be do better in a LIC, with good arguments both ways, but students’ drive to learn (as opposed to being taught) and independence in learning (ability to drive their own learning) are probably most important [60, 100].

There is some variation in the selection criteria where LICs are voluntary. The University of Alberta requires a good academic record and excludes students who have needed remediation in the past [99], while Minnesota selects on the basis of ‘reasonable academic achievement’ and the program’s ability to ‘match an individual student’s educational and geographic interests and needs’ [54]. A new program in Scotland does not exclude students on the basis of previous poor academic performance, as the continuity of learning relationships is seen as potentially beneficial to these students [51]. Stellenbosch University uses a range of factors including academic score, rural inclination, evidence of social responsibility, as well as tests of resilience and self-determination [101]. Generally, the focus is on self-directedness. Norris et al. [1] recommend that local site faculty are involved in student selection.

Some authors have attempted to define which students are likely to fit well into the learning environment of a LIC program. When considering the reasons for success in this specific aim rather than that of the sustainability overall, Halaas et al. [54] comment that students’ choices are ‘one factor’; those that are already interested in a rural, community-based career are more likely to choose the program. The concept of a ‘rural pipeline’ builds on this idea; where students with rural backgrounds are recruited into rurally based programs, it is likely that they will choose rural careers [102]. However, this should not be taken to mean that students without a rural background should be denied the opportunity to learn in a rural program, or that students from rural backgrounds would not do well in a LIC based in an urban setting. Brooks et al. [81] studied the characteristics of six cohorts of students on the University of Minnesota’s LIC program and concluded that the LIC students were ‘high achieving, self-directed and socially responsible individuals’ and that these characteristics may have been influenced by their rural backgrounds. They suggest that using personality profiling tools could help to match students to sites and guide mentoring arrangements.

A collaborative interview-based study among three medical schools in the USA aimed to identify and compare student and tutor descriptions of ‘the ideal medical student’ for LICs and block rotation curricula. In all three institutions, the LIC was an option for students rather than being compulsory [103]. This work suggests that LIC students placed greater emphasis on taking initiative and having responsibility for their own learning, saw themselves more strongly as caregivers as well as learners, expressed less pressure to perform and more willingness to admit what they did not know, and were less focused on being ‘a team player’ than were those on a block rotation. The authors interviewed participants early and late in the LIC and block rotation periods; because of differences in the responses at the beginning and the end of the year, they suggest that the differences in the learning environment experienced by the two groups were not wholly responsible for the differences in the students’ ideas, but that students selected the educational model that suits them best. Supervisors emphasized the need for LIC students to have more initiative, be more assertive and take greater ownership than students on a block rotation. They also identified more strongly the need for LIC students to be organized, able to prioritize and multitask, but did not highlight the care-giving role.

Konkin and Suddards [99] undertook an interview-based study at the University of Alberta whose voluntary rural LIC started in 2007. They concluded that students who choose a LIC should appreciate the importance of relationships and be able to develop them, be assertive in seeking out learning opportunities and social interactions, be able to adapt to different environments and be able to see the benefits of their experiences.

While there are several studies that have considered the question of which students do best on a LIC, there is no consensus in the literature. It is not yet possible to predict those characteristics of students which would result in them thriving or being challenged within a LIC curriculum. With the increase in the number of ‘whole cohort’ LICs, there may be opportunities to undertake this research and cross-institutional collaborations may be helpful.

#### Guideline 15: *How is cost-effectiveness best measured?*

In order to achieve sustainability, a LIC’s required resources must be identified, acknowledged and met. Some groups have attempted to quantify or model the financial costs [26, 28, 51, 86, 104] but there is no consistent method of costing them. For many LICs in new locations, costs are more explicit than they are in previously existing programs, where costs may be more difficult to identify in tertiary teaching hospital budgets or university staffing arrangements. This may result in the costs for LICs appearing to be higher than more traditional programs. Norris et al. [1] noted that in 2009 reliable data on costs were not available. Norris et al. [1] and Worley et al. [2] acknowledge the uncertainty about the cost-effectiveness and sustainability of the LIC model in terms of a broad range of outcomes.

Any attempt to define cost-effectiveness must take into account the context of the economic benefit to communities; as well as the direct contributions of students to the local economies, there are broader beneficial effects for communities over time [105]. These include increases in the attractiveness of communities to other professionals and businesses and more investment in infrastructure, partly as a result of the presence of a medical school (or medical learners) and partly as a result of it being better provided with healthcare in the longer term as recruitment improves.

A consistent measure of cost-effectiveness in relationship to defined outcomes would help in future comparative work.

#### Guideline 16: *What is the optimal structure in a LIC timetable?*

Most LICs involve a mixture of a structured timetable and ‘white space’ so that students can see patients, follow their healthcare journeys and find opportunities to meet their own self-directed learning needs associated with the patients they have encountered. There is variability between LIC programs in how much guidance is given in terms of the number and characteristics of the patients that students are expected to follow over time. Some LICs involve structured groups of patients with quite a lot of input from faculty regarding their composition, whereas others are more student-led. In a number of North American programs, accreditation requirements influence the composition of students’ patient groups.

The structured timetable varies considerably by program; for some the majority of the structured time is in general practice/family medicine while for others it is in hospital-based specialty clinics. Perhaps the key issue is that LICs are inherently flexible and adaptable to context; within the LIC of a single institution there could be flexibility for distributed sites to adapt the structure as they and their students see fit, as long as the overall curricular goals are met.

Owen et al. [106] and Levitt et al. [74] consider the question of learning maps and trajectories as frameworks for learning in LICs. The impacts of these on students’ learning is not known.

#### Guideline 17: *How long should a LIC be?*

Despite the focus on continuity of care and of learning relationships, there is no generally accepted length for a LIC; Worley et al. [2] report a range of 6–54 weeks with a median length of 40 weeks, though not all of the programs included fell within the definition of LICs. The question of how much time it takes to achieve the goals of a LIC was raised in this paper and remains unanswered in the published literature to date.

Given that the effectiveness of LICs is considered to depend on relationships in communities of practice, they need to be long enough for students to become legitimate contributors to healthcare within them and for the community of practice itself to be able to adapt to the cycle of students joining and leaving it.

There is a general sense in the literature that ‘more is better’ [5, 107, 108] and that the benefits to students and faculty, and recruitment to community-based careers, may be greater from longer placements [67].

Worley and Kitto [86] concluded that at least 5 or 6 months would be required for the economic sustainability of a LIC based in a rural setting in Australia.

Crampton et al. [108] suggest 14 weeks may be enough for students and tutors to develop effective learning relationships in the context of extended placements in a deprived urban area in the UK and Bartlett et al. [42] found that a 15 week extended placement (an amalgamative LIC by Worley et al.’s criteria [2]) in an English rural setting had the same effect and was enough to lead some students to consider a rural career.

We are not aware of any literature which addresses the question of how long would be too long, though in 2013, a review of the LIC literature concluded that ‘a whole year is definitely long enough’ [109]. Since then, O’Sullivan et al. [93] compared 1 and 2 year immersive learning in rural settings in Australia with regard to graduates’ future career choices. They found that the strongest benefit on early career doctors choosing rural areas occurred where students had a mixture of rural general practice and regional hospital experience, and this was incremental up to a length of 3 years of undergraduate placements. These students were not on a LIC program for the whole of this time [93].

The question of an ideal length may not be answerable but it is one which has been asked in the literature and may merit further exploration. The existing literature demonstrates that the LICs involved may be long enough for students to develop effective learning relationships, practice patient-centred care and succeed in assessments but there is not a consensus about a minimum length to guarantee this. The evidence for how long a LIC would need to be to influence career choices or to maximize cost-effectiveness is similarly inconclusive. Future research will need to specify which outcomes are being considered.

#### Guideline 18: *What are the effects on patient care?*

Part of a consideration of the sustainability of any healthcare service must be its impact on its users. To be sustainable it must be acceptable to them, meet their needs, lead to better outcomes and not cause them harm. The distribution of the workforce to areas of need will lead to health benefits for communities and it is known that LIC programs have positive impacts on that [10].

At an individual patient level there is evidence that patients’ experiences of healthcare are enhanced by the continuity of relationships with LIC students (see Guideline 4).

Work by Hudson et al. [61] in Australia demonstrated that patients found their involvement with LIC students acceptable and they identified new roles for themselves as partners in medical education in communities of practice and the development and potential recruitment of new doctors in their communities. They reported that they learnt about their illnesses, shared in decision making, and had more autonomy as a result of working with the students.

Poncelet et al. [110] found that patients in an urban setting valued their interactions with LIC students over time. They perceived that the students took on ‘physician-like’ roles and as a result described enhanced access to and coordination of care, communication, patient education and wellbeing. In particular, they described the students as acting as a bridge between themselves and their physicians.

It is known that students who learn in LIC structures do at least as well in assessments and often better than their peers on block rotations [29, 111], that they are likely to be more patient-centred [10, 54], ready to act as the patient’s advocate and be motivated by an ‘ethic of caring’ [52], and that these effects endure after graduation [91] but we are not aware of any published evidence that this translates into measurable benefits to patients in terms of their health and well-being. This may be because of difficulties in measuring these benefits and isolating the causes of variations over time.

## Conclusion

The question of what makes a complex program sustainable is a difficult one to answer and this is compounded here by the variety of different models of LICs worldwide and their relatively recent adoption in many cases. However, this narrative review, which has drawn on our experience of setting up and evaluating LICs over four continents and their familiarity with the literature, has resulted in a degree of consensus on some of the questions which may provide some guidance for others. The list of Dos, Don’ts and Don’t Knows effectively provides a summary of our review. Perhaps the main points that need to be made are that the complexity of the change management processes requires careful and sustained attention, and that there is a need to support students and faculty though them and beyond into an established LIC. There is an increasing body of clear evidence of the benefits of doing so.
