# Correction to: The do’s, don’ts and don’t knows of establishing a sustainable longitudinal integrated clerkship

**DOI:** 10.1007/s40037-020-00570-8

**Published:** 2020-02-27

**Authors:** Maggie Bartlett, Ian Couper, Ann Poncelet, Paul Worley

**Affiliations:** 1 Education in General Practice, Dundee University School of Medicine, Dundee, UK; 2 Faculty of Medicine and Health Sciences , Ukwanda Centre for Rural Health, Stellenbosch University, Stellenbosch, South Africa; 3 Department of Neurology, University of California, San Francisco, CA USA; 4 Department of Health, GPO Box 9848, 2601 Canberra, Australian Capital Territory Australia; 5 Prideaux Centre for Research in Health Professions Education, Flinders University, Adelaide, Australia

**Correction to:**

**Perspect Med Educ 2019**

10.1007/s40037-019-00558-z

Unfortunately information regarding Paul Worley’s affiliation is missing from the original article. Please find the information here:

Paul Worley is affiliated to the Prideaux Centre for Research in Health Professions Education, Flinders University, Adelaide, Australia. He is the Australian National Rural Health Commissioner. This is an independent statutory position. The views in this paper are those of the authors only and do not represent the views of the Australian Government or the Department of Health.

